# Deciphering the Mechanisms Shaping the Plastisphere Microbiota in Soil

**DOI:** 10.1128/msystems.00352-22

**Published:** 2022-07-26

**Authors:** Yuanze Sun, Jia Shi, Xiang Wang, Changfeng Ding, Jie Wang

**Affiliations:** a Beijing Key Laboratory of Farmland Soil Pollution Prevention and Remediation, College of Resources and Environmental Sciences, China Agricultural University, Beijing, China; b College of Land Science and Technology, China Agricultural University, Beijing, China; c Key Laboratory of Soil Environment and Pollution Remediation, Institute of Soil Science, Chinese Academy of Sciences, Nanjing China; University of Illinois at Chicago

**Keywords:** assembly processes, microplastics, network, plastisphere, soil microbiology

## Abstract

The gradual accumulation of microplastics has aroused increasing concern for the unique niche, termed “plastisphere.” As research so far has focused on their characteristics in aquatic ecosystems, our understanding of the colonization and assembly of the attached bacterial communities on microplastics in soil ecosystems remains poor. Here, we aimed to characterize the plastisphere microbiomes of two types of microplastics (polylactic acid [PLA] and polyethylene [PE]) differing in their biodegradability in two different soils. After incubation for 60 days, considerably lower alpha diversity of bacterial community was observed on the microplastic surfaces, and prominent divergences occurred in the microbial community compositions between the plastisphere and the bulk soil. The temperature, rather than polymer type, significantly induced the differences between the plastisphere communities. The rRNA gene operon (*rrn*) copy numbers were significantly higher in the PLA plastisphere, suggesting potential degradation. The co-occurrence network analysis showed that the PE plastisphere exhibited greater network complexity and stronger stability than those in the PLA plastisphere. The stochasticity ratio indicated the remarkable importance of stochastic process on community assembly in PE and PLA plastispheres, while the null model analysis showed the nonnegligible roles of deterministic processes in shaping the plastisphere communities. Higher contributions of homogenous selection in the PLA plastisphere were observed in comparison with the PE plastisphere, which could probably be attributed to the selective pressure induced by microplastic degradation. Our findings enhance our mechanistic understanding of the diversity patterns and assembly processes of plastisphere in soil environments and have important implications for microbial ecology and microplastic risk assessment.

**IMPORTANCE** The increasing pervasive microplastic pollution is creating a new environmental compartment, termed plastisphere. Even though there was conclusive information characterizing the plastisphere, the underlying mechanisms shaping the bacterial communities in the plastisphere in the soil remain unclear. Therefore, we incubated two types of microplastics (PE and PLA) in two different soils and explored the differences between plastisphere and bulk soil communities. Additionally, the co-occurrence network and the assembly processes of plastisphere were subjected to further analysis. Our results highlight the importance of selective recruitment of microplastics and contribute to the understanding of the diversity patterns and assembly processes of plastisphere in soil environments.

## INTRODUCTION

Global plastic production has enormously increased from 1.5 million tons in 1950 to 367 million tons in 2020 because of its durability, malleability, and low cost (1). Despite the remarkable benefits and convenience of plastics to human lives, there is increasing awareness about the negative environmental impacts arising from the vast amount of plastic waste (2, 3). Due to the environmental weathering over time, fragmentation of plastics can happen, leading to microplastic (<5 mm) generation (4). Microplastics have been ubiquitously detected in various environments, such as oceans (5, 6), rivers (7, 8), lakes (9, 10), sediments (11, 12), and soils (13, 14). While their abundance and subsequent effects in the aquatic environments have been found to be of great importance and have been studied already for a decade, their occurrence and potential impact on the soil ecosystem have only been recognized more recently (15). Microplastics can enter soils through a range of routes, including atmospheric deposition (16), plastic mulching application (17), organic manure fertilization (18), and water irrigation (19). The presence and persistence of microplastics in soils have raised significant concerns as adverse biological effects across trophic levels and impacts on ecosystem function are being documented (20).

As an exogenous and hydrophobic substrate, microplastic surfaces can provide a unique niche for the growth and proliferation of a diversity of microorganisms, constituting a distinct ecological habitat called the “plastisphere” (21–23). The microbial assemblages in the plastisphere have usually been reported to differ in taxonomic composition and structure from those in the surrounding natural media. Studies also suggested that the plastisphere may contain potential invasive and pathogenic species (24). Through metagenomic sequencing, Bhagwat et al. reported that, along with exhibiting unique microbial profiles, the plastisphere specifically enriched the functions related to xenobiotic compound degradation, carbon cycling, and plant-pathogen interaction (25). Additionally, the plastisphere has been considered a hot spot for horizontal gene transfer, potentially facilitating the transfer of pathogenicity and resistance in the environment (26). Despite conclusive information characterizing the plastisphere, most studies have only been focused on the aquatic environment. Data about the plastisphere microbiome in soil ecosystems are scarce. The mechanisms shaping the community diversity and composition of plastisphere in soils remain unclear.

The dominance of stochastic processes in driving plastisphere bacterial communities was observed in aquatic environments, as the polymer types, sampling sites, exposure time, and, more importantly, mobility in water may introduce high stochasticity (27, 28). In comparison with aquatic environments, the microplastic transportability in soils was relatively low. The importance of ecological processes in shaping soil plastisphere may be largely different from those in aquatic environments. Additionally, the soil is not a matrix with a uniform temperature. Climate warming can alter soil microbial community diversity, structure, and activities (29, 30), but it remains uncertain whether and how warming impacts the plastisphere characteristics and its assembly mechanisms.

To address the knowledge gap, this study conducted a microcosm experiment with two different microplastic types in two different soils at 15 and 25°C, respectively. We used polyethylene (PE) and polylactic acid (PLA) microplastics because they may recruit specific microbial taxa due to their different biodegradability. Our main questions are the following. (i) To what extent is the soil plastisphere presented in a distinctive manner from the microbial communities in soil particles? (ii) Does temperature or plastic polymer have a significant impact on the characteristics of plastisphere in soil? (ii) What are the relative roles of deterministic and stochastic processes in shaping the microbial communities on soil microplastics?

## RESULTS AND DISCUSSION

### Comparison of plastisphere and soil bacterial communities.

**FIG 1:**
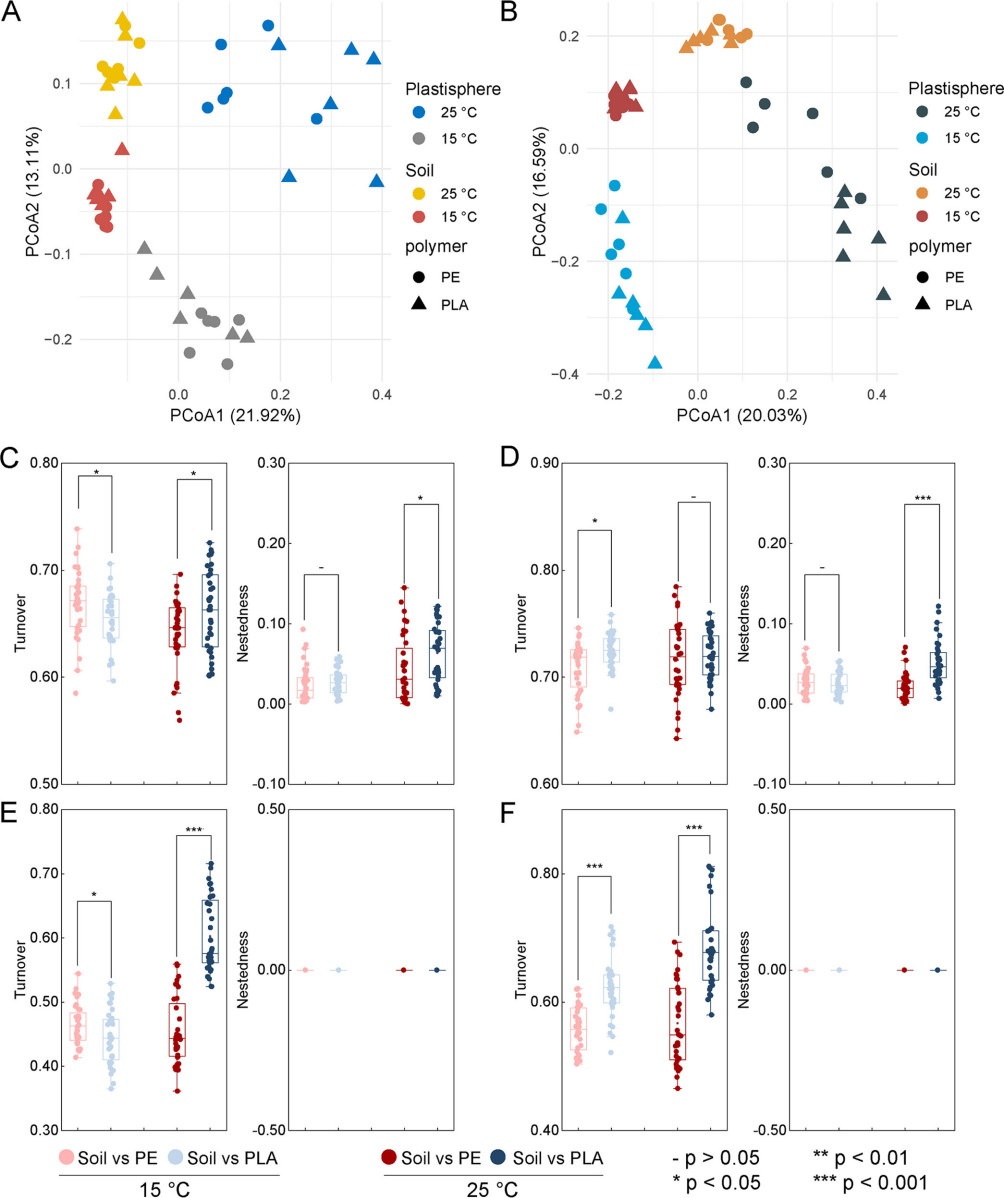
Beta diversity of plastisphere and bulk soil bacterial communities. (A) Principal-coordinate analysis (PCoA) based on Bray-Curtis dissimilarity showing the beta diversity of plastisphere and bulk soil bacterial communities in BS treatments. (B) PCoA based on Bray-Curtis dissimilarity showing the beta diversity of plastisphere and bulk soil bacterial communities in YS treatments. (C) Differences in turnover and nestedness on the Jaccard distances-based beta diversity patterns of bacterial communities between plastisphere and bulk soil in BS treatments. (D) Differences in turnover and nestedness on the Jaccard distances-based beta diversity patterns of bacterial communities between plastisphere and bulk soil in YS treatments. (E) Differences in turnover and nestedness on the Bray distances-based beta diversity patterns of bacterial communities between plastisphere and bulk soil in BS treatments. (F) Differences in turnover and nestedness on the Bray distances-based beta diversity patterns of bacterial communities between plastisphere and bulk soil in YS treatments.

The principal-coordinate analysis (PCoA) and permutational multivariate analysis of variance (PERMANOVA) analysis of all blank clay soil (BS) samples on the basis of Bray-Curtis dissimilarity suggested that the variation in the bacterial community was mainly explained by the compartment niche (*R*^2^ = 17.0%, *P < *0.001) and temperature (*R*^2^ = 12.5%, *P < *0.001) (Fig. 1A; see Table S2 in the supplemental material), while for yellow loam sand soil (YS) samples, the variation was primarily explained by the temperature (*R*^2^ = 17.6%, *P < *0.001), followed by the niche (*R*^2^ = 13.7%, *P < *0.001) (Fig. 1B; Table S2). Similar results were also observed on the basis of Jaccard distances (Fig. S1). The findings indicated that the plastisphere communities in soil environments were significantly distinct from the bulk soil microbial communities, which was consistent with the general observation in aquatic environments (21–24, 26). The difference between the plastisphere and bulk soil was further influenced by the temperature. Higher temperature (25°C) significantly increased the dissimilarity between the PLA plastisphere and the soil bacterial community, whereas no effects were observed for the PE plastisphere (Fig. S1). The shifts between niches can be driven by turnover (the replacement of species between plastisphere and bulk soil) or nestedness (the subsetting of communities between niches) (31). Thus, the contribution of turnover and nestedness to the dissimilarity between plastisphere and bulk soil was partitioned based on Jaccard and Bray-Curtis distances (Fig. 1C through F). For all measures, turnover dominated the dissimilarity between the plastisphere and bulk soil compared to nestedness. Additionally, the contribution of nestedness was higher for the dissimilarity between the PLA plastisphere and the soil community at 25°C.

10.1128/msystems.00352-22.2 TABLE S2 Effects of niche, temperature, and polymer type on bacterial community structure in different soil incubations based on PERMANOVA. Download Table S2, DOCX file, 0.01 MB. Copyright © 2022 Sun et al. 2022 Sun et al. https://creativecommons.org/licenses/by/4.0/ This content is distributed under the terms of the Creative Commons Attribution 4.0 International license.

10.1128/msystems.00352-22.5 FIG S1 Beta diversity and dissimilarity of plastisphere and bulk soil bacterial communities. Principal-coordinate analysis (PCoA) based on Jaccard dissimilarity showing the beta diversity of plastisphere and bulk soil bacterial communities in BS (A) and YS (B) treatments. (C and D) Dissimilarity between plastisphere and bulk soil communities based on Bray-Curtis distances in BS (C) and YS (D) treatments. (E and F) Dissimilarity between plastisphere and bulk soil communities based on Jaccard distances in BS (E) and YS (F) treatments. Download FIG S1, PDF file, 0.3 MB. Copyright © 2022 Sun et al. 2022 Sun et al. https://creativecommons.org/licenses/by/4.0/ This content is distributed under the terms of the Creative Commons Attribution 4.0 International license.

**FIG 2:**
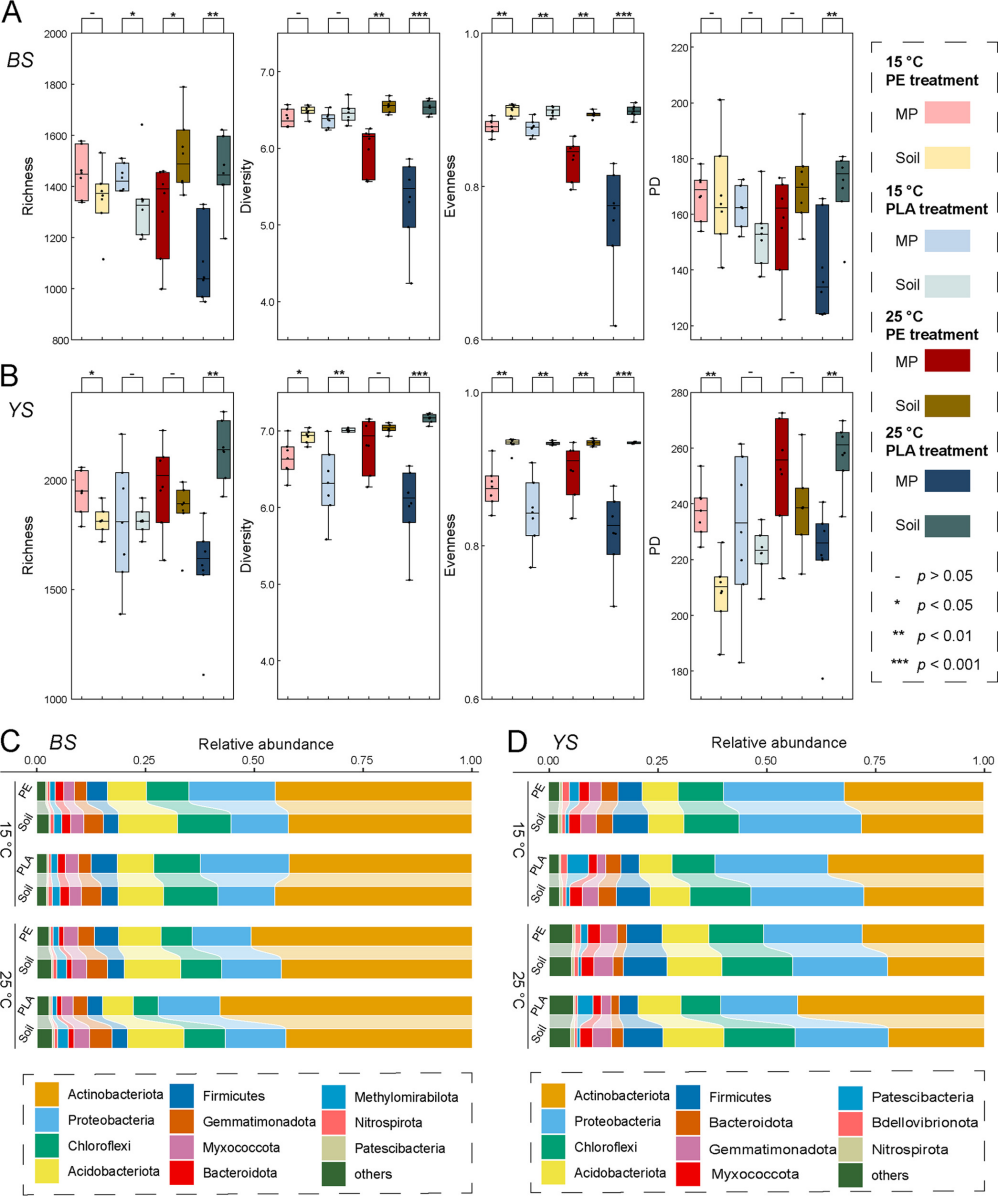
Alpha diversity and community composition of plastisphere and bulk soil bacterial communities. (A) Alpha diversity indices (Chao1 richness, Shannon diversity, Pielou’s evenness, and Faith’s phylogenetic diversity) of plastisphere and bulk soil bacterial communities in BS treatments. (B) Alpha diversity indices (Chao1 richness, Shannon diversity, Pielou’s evenness, and Faith’s phylogenetic diversity) of plastisphere and bulk soil bacterial communities in YS treatments. (C) Relative abundances of the main phyla in BS treatments. (D) Relative abundances of the main phyla in YS treatments.

Using several alpha diversity estimators, we found that the plastisphere generally harbored lower bacterial richness, lower diversity, and reduced evenness than the bulk soils at 25°C (Fig. 2A and B), while for the incubation at 15°C, the variations in alpha diversity between plastisphere and bulk soil were close. The dominant bacteria phyla in the BS plastisphere were *Actinobacteriota*, *Proteobacteria*, *Acidobacteriota*, *Chloroflexi*, and *Firmicutes* (Fig. 2C). In comparison with the soil communities, the relative abundances of *Acidobacteriota*, *Chloroflexi*, *Gemmatimonadota*, and *Methylomirabilota* were relatively lower in PE and PLA plastispheres (Fig. 2C; Fig. S2). For instance, the relative abundances of the phylum *Acidobacteriota* in the plastisphere ranged from 7.05% to 13.0%, while the abundances in the soil were from 8.47% to 13.6%. *Actinobacteriota* and *Proteobacteria* showed greater abundance in the plastisphere, and high temperature significantly increased the relative abundance of *Actinobacteriota* in the plastisphere and decreased the values of *Proteobacteria*. At 25°C, the abundances of *Actinobacteriota* in PE and PLA plastisphere were 58.1% and 50.8%, respectively, which were significantly higher than the values at 15°C (45.1% and 41.9%). For the phylum *Proteobacteria*, the abundances in PE and PLA plastisphere at 25°C were 13.5% and 14.1%, respectively, whereas the abundances increased to approximately 20% at 15°C. Similar to the BS samples, the most dominant phyla in YS samples were also *Actinobacteriota*, *Proteobacteria*, *Chloroflexi*, *Acidobacteriota*, and *Firmicutes* (Fig. 2D). The relative abundances of *Chloroflexi*, *Firmicutes*, *Gemmatimonadota*, and *Nitrospirota* in PE and PLA plastisphere were significantly lower than the values in the soils, whereas the levels of *Actinobacteriota*, *Patescibacteria*, and *Bdellovibrionota* were statistically higher (Fig. 2D; Fig. S2).

10.1128/msystems.00352-22.6 FIG S2 Comparison of the main phyla in plastisphere and bulk soil communities in BS (A) and YS (B) treatments at different temperatures. Download FIG S2, PDF file, 0.6 MB. Copyright © 2022 Sun et al. 2022 Sun et al. https://creativecommons.org/licenses/by/4.0/ This content is distributed under the terms of the Creative Commons Attribution 4.0 International license.

The beta diversity represents the dissimilarity in species composition between distinct locations, and it consists of turnover and nestedness. Species turnover means species are replaced by a different species along spatial gradients, whereas nestedness is a nonrandom process of species disappearance or emergence (31). In the current study, turnover was a much greater contributor to the beta diversity between bulk soil and plastisphere communities, reflecting the high proportion of shared species present in both bulk soil and plastisphere communities. Additionally, only species turnover cannot explain all differences between soil and plastisphere communities, especially for the PLA plastisphere at 25°C. The contribution of nestedness to the total dissimilarity between PLA plastisphere and bulk soil communities ranged from 1.4% to 16.3%. In comparison with PE microplastics, the PLA has greater microbial degradability. Therefore, the PLA plastisphere may particularly recruit the species that can utilize the oligomers as carbon sources, while for PE treatments, nestedness also contributed to the differences between bulk soil and plastisphere communities. This was probably attributed to the high hydrophobicity of PE polymer, which may encourage the microplastics to sorb dissolved organic matters and recruit copiotrophic bacteria. The deflective selection may lead to species loss in the plastisphere and also contribute to the relatively lower levels of alpha diversity in plastisphere communities. The selective recruitment also referred to the different bacterial community compositions. *Actinobacteriota* and some *Proteobacteria* were generally considered to be more copiotrophic (32, 33) and showed greater relative abundances in the plastispheres. In contrast, *Acidobacteria* and *Gemmatimonadota* were well adapted to the oligotrophic condition (34), which had relatively lower abundances in plastisphere.

### Comparison of PE and PLA plastispheres.

**FIG 3:**
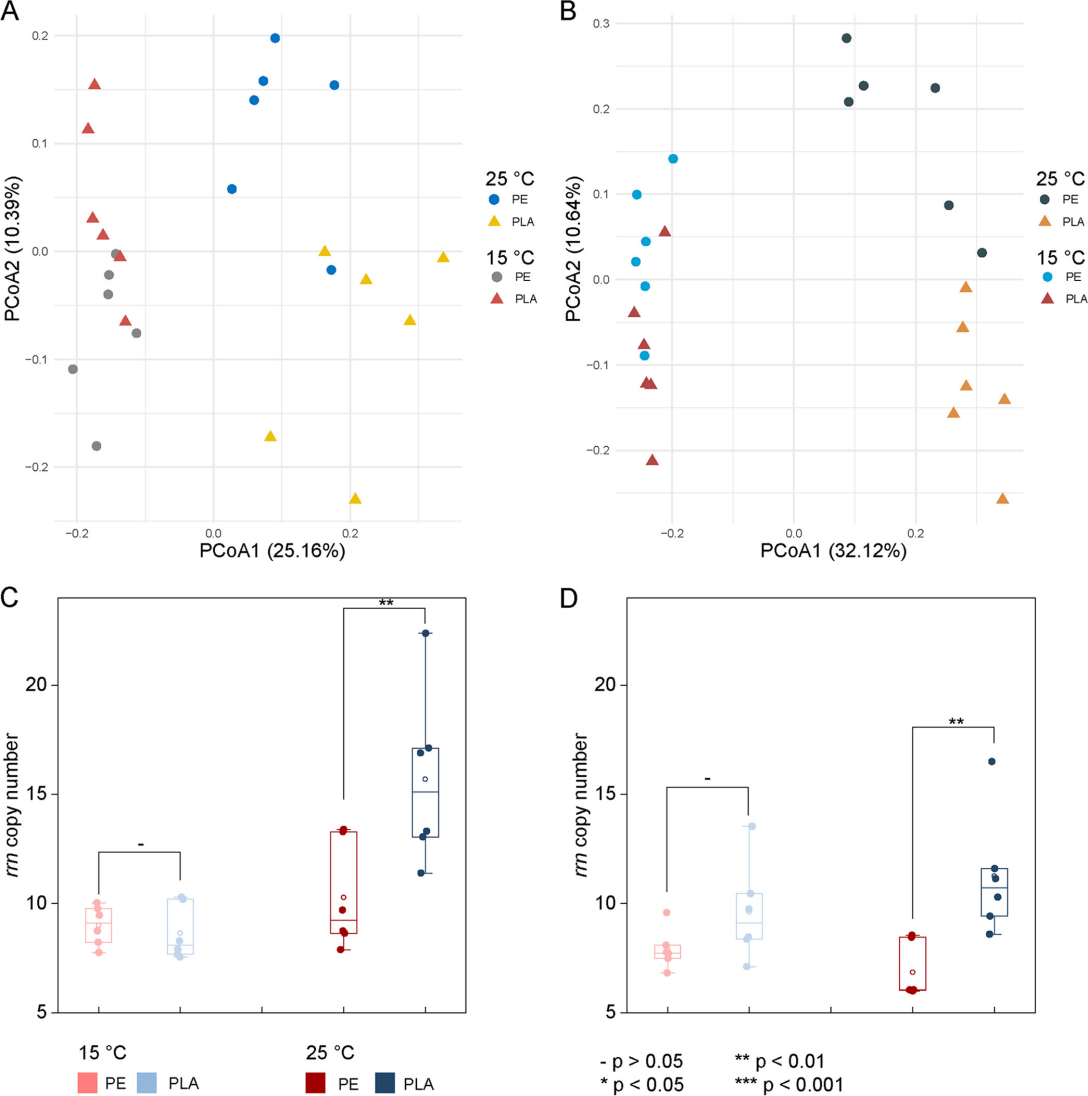
Comparison between PE and PLA plastispheres. (A) Principal-coordinate analysis (PCoA), based on Bray-Curtis dissimilarity, showing the beta diversity of PE and PLA plastispheres in BS treatments. (B) PCoA based on Bray-Curtis dissimilarity showing the beta diversity of PE and PLA plastispheres in YS treatments. (C) rRNA gene operon (*rrn*) copy number in plastisphere bacterial communities in BS treatments. (D) *rrn* copy number in plastisphere bacterial communities in YS treatments.

The relative abundances of the main phyla in the plastisphere show minimal obvious differences between PE and PLA. Only significant temperature variations in the composition of plastisphere bacterial communities were observed (PERMANOVA, *P* = 0.001) (Table S3). Even though a separating trend between PE and PLA plastispheres can be observed along the second PCoA axes for both BD and YS treatments (Fig. 3A and B; Fig. S3), the PERMANOVA results indicated that polymer type did not show a significant influence on plastisphere bacterial communities. For the alpha diversity, there was no difference between PE and PLA plastispheres at 15°C incubation, whereas the PLA plastisphere showed a significantly lower level of alpha diversity than the PE plastisphere at 25°C (Fig. 2). For instance, the richness values of PE plastisphere in BS and YS treatments were 1,465 and 1,969, respectively, which was obviously higher than the values in the PLA plastisphere (1,106 and 1,587, respectively). The deflective selection of oligomer-degraded bacteria on PLA microplastics may refer to the lower alpha diversity.

10.1128/msystems.00352-22.3 TABLE S3 Effects of temperature and polymer type on plastisphere community structure in different soil incubations based on PERMANOVA. Download Table S3, DOCX file, 0.01 MB. Copyright © 2022 Sun et al. 2022 Sun et al. https://creativecommons.org/licenses/by/4.0/ This content is distributed under the terms of the Creative Commons Attribution 4.0 International license.

10.1128/msystems.00352-22.7 FIG S3 (A) Principal-coordinate analysis (PCoA) based on Jaccard dissimilarity showing the beta diversity of PE and PLA plastispheres in BS treatments. (B) PCoA based on Jaccard dissimilarity showing the beta diversity of PE and PLA plastisphere in YS treatments. (C) Indicator species (blue blocks) in plastisphere communities in BS treatments. The four circles from inner to outer are PE plastisphere at 15°C, PLA plastisphere at 15°C, PE plastisphere at 25°C, and PLA plastisphere at 25°C. (D) Indicator species (blue blocks) in plastisphere communities in YS treatments. The four circles from inner to outer are PE plastisphere at 15°C, PLA plastisphere at 15°C, PE plastisphere at 25°C, and PLA plastisphere at 25°C. Download FIG S3, PDF file, 0.7 MB. Copyright © 2022 Sun et al. 2022 Sun et al. https://creativecommons.org/licenses/by/4.0/ This content is distributed under the terms of the Creative Commons Attribution 4.0 International license.

We further employed indicator species analysis to identify the amplicon sequence variants (ASVs) whose abundances were significantly greater in the plastisphere than the respective soil communities (Fig. S3). A total of 186 ASVs were identified as the indicator species in the BS plastisphere, which were predominated by *Proteobacteria* (68), *Actinobacteriota* (50), *Myxococcota* (14), *Chloroflexi* (14), and *Firmicutes* (14). There were 100 and 71 indicator ASVs in the PE and PLA plastispheres at 15°C, while at 25°C, the values were 34 and 67. Six indicator ASVs were shared in all plastisphere samples in BS treatments, and they mainly belonged to the genera *Nocardia* and *Lysobacter*. In the YS plastisphere, a total of 311 indicator ASVs were obtained, which mainly belonged to the phyla *Proteobacteria* (104), *Actinobacteriota* (79), and *Patescibacteria* (24). In the PE plastisphere, there were 88 and 75 indicator ASVs at 15 and 25°C, respectively, while the PLA plastisphere had 128 and 158 indicators, respectively. Twelve indicator ASVs, belonging to the genera *Nocardia*, *Streptomyces*, and *Lysobacter*, were shared in all YS plastispheres. Previous studies also indicated that these genera were detected on microplastics. For instance, Yi et al. (35) observed that the abundance of *Lysobacter* was higher in membranous polypropylene than in the corresponding soils (35). The enrichment of *Streptomyces* and *Lysobacter* in the plastisphere may be related to their ability to degrade polymers and complex organic matters (36, 37). Additionally, *Nocardia* is mostly known for its ability to cause localized and systemic infections in humans (38). The enrichment of this genus potentially indicated that both conventional and biodegradable microplastics may act as the vector for transporting disease-causing bacteria.

To further illustrate the difference between PE and PLA plastispheres, the rRNA gene operon (*rrn*) copy number at the community level was calculated (Fig. 3C and D) (39). Previous studies indicated the *rrn* copy number at the species level was closely correlated with the growth rate and nutrient utilization efficiency of individual organisms, and the community-level *rrn* copy numbers were positively correlated with environmental nutrient contents (40, 41). The community *rrn* copy number for plastisphere in BS ranged from 7.7 to 22.4, while the values of plastisphere in YS ranged from 6.1 to 16.5. No significant difference was observed between PE and PLA plastispheres at 15°C, while the community-level *rrn* copy numbers of the PLA plastisphere were statistically higher than those in the PE plastisphere. As a high *rrn* copy number is favored in copiotrophic environments, we may conclude that the PLA supplied a nutrient-rich environment compared to PE at 25°C, potentially indicating the degradation of PLA microplastics. Additionally, greater variance in the community-level *rrn* copy numbers was observed for the PLA plastisphere at 25°C, potentially suggesting the heterogeneity of PLA degradation in the soil. In the current study, the air permeability and water conditions may vary across the whole bulk soil. Given the sensitivity of PLA degradation to water and temperature, the degree of PLA degradation may be different, thus inducing great variance in the community-level *rrn* copy number.

**FIG 4:**
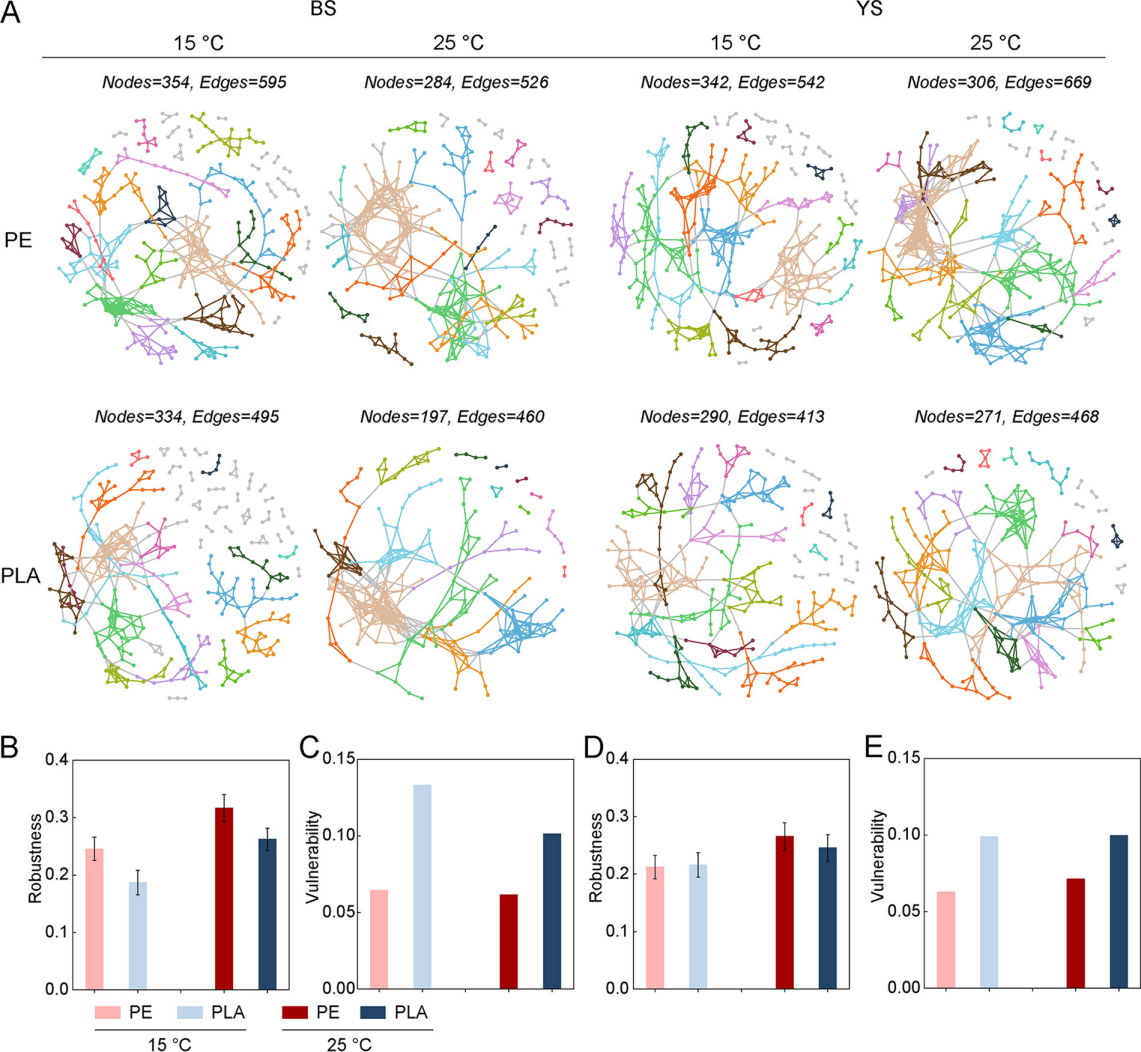
Network association of bacterial ASVs in each plastisphere. (A) Visualization of constructed network of PE and PLA plastispheres in BS and YS treatments at different temperature. Large modules are shown in different colors, and smaller modules are shown in gray. (B) Robustness measured as the proportion of taxa remained with 50% of the taxa randomly removed from each plastisphere network in BS treatments. (C) Network vulnerability of plastisphere in BS treatments. (D) Robustness measured as the proportion of taxa remained with 50% of the taxa randomly removed from each plastisphere network in YS treatments. (E) Network vulnerability of plastisphere in YS treatments.

The changes in microbial network structure affected community functioning and stability. Therefore, we constructed random matrix theory-based networks for all plastispheres to evaluate the potential ecological interactions among bacterial members (Fig. 4A). All the networks exhibited scale-free, small-world, and nonrandom characteristics (*R*^2^ of power law, 0.698 to 0.838). Fewer nodes and links were observed in the plastisphere networks at 25°C than those at 15°C, but these networks showed higher levels of average degree (avgK) and average clustering coefficient (avgCC) and lower average path distance (GD), potentially suggesting more densely connected groups on microplastics at higher temperature (Table S4). We further calculated the robustness (the resistance to node loss) of the networks (Fig. 4B and D). After the removal of random species, the networks had significantly higher robustness at 25°C than those at 15°C, indicating higher stability in warming networks. PE plastisphere networks showed greater complexity than PLA plastisphere networks. For instance, the node and link numbers in PE plastisphere networks ranged from 284 to 354 and 526 to 669, respectively, which were higher than the values in PLA plastisphere networks. A recently developed metric, called cohesion, was calculated to quantify the connectivity of the plastisphere community, and it can also reflect the degree of community complexity (42). Positive cohesion could reflect the degree of cooperative behaviors, with greater values indicating closer cooperation among bacterial members. Negative cohesion could indicate the magnitude of competitive behaviors, with higher absolute values signifying more competition among bacterial members. Even though there were no statistical differences between PE and PLA plastisphere networks, higher levels of positive and negative cohesion in PE plastisphere networks were observed, suggesting that the potential biotic interactions in PE were relatively stronger (Fig. S4). Greater robustness was observed in PE plastisphere networks; simultaneously, the PE network vulnerability was lower than the PLA plastisphere networks, potentially indicating that the microbial community may be more stable in PE than that in PLA (Fig. 4C and E).

10.1128/msystems.00352-22.4 TABLE S4 Topological properties of plastisphere networks in BS and YS treatments. Download Table S4, DOCX file, 0.02 MB. Copyright © 2022 Sun et al. 2022 Sun et al. https://creativecommons.org/licenses/by/4.0/ This content is distributed under the terms of the Creative Commons Attribution 4.0 International license.

10.1128/msystems.00352-22.8 FIG S4 (A) Positive cohesion of plastisphere networks in BS treatments. (B) Positive cohesion of plastisphere networks in YS treatments. (C) Negative cohesion of plastisphere networks in BS treatments. (D) Negative cohesion of plastisphere networks in YS treatments. Download FIG S4, PDF file, 0.2 MB. Copyright © 2022 Sun et al. 2022 Sun et al. https://creativecommons.org/licenses/by/4.0/ This content is distributed under the terms of the Creative Commons Attribution 4.0 International license.

The observation that higher temperature reduced the numbers of network nodes and links may be attributed to the decreased alpha diversity in plastisphere communities. As shown in Fig. 2, the richness and diversity in plastisphere communities were obviously higher at 15°C than those at 25°C. Previous studies have indicated that higher temperature may induce the hydrolyzation of PLA microplastics. Therefore, at higher temperature, the oligomers from PLA microplastics may act as the selective carbon source and filter microbial species with special functions (e.g., degrading oligomers), which, in turn, decreases the alpha diversity, while PE microplastics were not biodegradable but could sorb the dissolved nutrient from the soil due to their high hydrophobicity. Higher temperature may enhance this process, which may also act as the filtering factor and reduce the alpha diversity. On the other hand, the rising temperature would stimulate various biotic interactions because of the faster growth and more active individual metabolic processes. Therefore, in our study, the higher temperature reduced the number of microbial species involved in the networks but increased their interactions. Additionally, the distinct biodegradation between PLA and PE microplastics also resulted in higher *rrn* copy numbers and lower alpha diversity in the PLA plastisphere, which may be the reason that PLA plastisphere networks were less complex and stable than PE plastisphere networks.

Whether microplastics could really select specific microbial communities remains questionable. Wright et al. have reviewed the studies about plastisphere communities in aquatic environments and stated that the planktonic microbes would strongly diverge from those able to develop biofilms (43). They also declared that the plastisphere was just a biofilm, but on microplastics, and may have similar communities to those on other material surfaces. In comparison with planktonic bacteria free-living in water, soil bacteria are generally associated with natural material particles. Considering our results that the community compositions between plastisphere and bulk soil were significantly different, we may conclude that the plastisphere exhibits distinct bacterial communities from those in the surrounding environments. Simultaneously, the mechanisms induced by the distinct communities in the plastisphere may be different between PE and PLA microplastics. PE is a typical nonbiodegradable petroleum-based polymer, but with great hydrophobicity, which can sorb nonpolar organic matters and thus recruit copiotrophic species. PLA is a polyester made from the polymerization of lactic acid, which can be naturally degraded by the simple hydrolysis of ester bonds. The produced low-molecular-weight oligomers may recruit degrading bacteria and lead to community convergence. The divergence in degradation between PE and PLA also contributed to the distinct properties of plastisphere networks.

### The potential sources and assembly processes of the plastisphere microbiome.

Identifying the potential origins of the plastisphere microbiome may be primary for exploring their assembly processes. Therefore, a source-tracking analysis was conducted to identify the contribution of bulk soil microbial communities to the plastisphere microbiomes (Fig. S5). The results suggested the plastisphere ASVs mainly derived from bulk soils, ranging from 19.9% to 95.9%. In BS soil, the contribution of soil communities to the PE plastisphere at 25°C was significantly higher than those to the PLA plastisphere. Similarly, the relative contribution of bulk soil to PE plastisphere in YS soil ranged from 57.5 to 95.5%, which was significantly higher than the contribution to the PLA plastisphere. The high contribution of bulk soil may be expected due to the static incubation systems in the soil. In contrast, when the microplastics were incubated in aquatic environments, the free-living microorganisms in the surrounding water may have been constantly in motion. Thus, the contribution of free-living microorganisms to the plastisphere in the aquatic environment should be lower. To verify our hypothesis, the sequencing data in our previous studies, which compared the planktonic and plastisphere communities in urban rivers and estuaries, were further analyzed to identify the contribution of free-living bacteria to the plastisphere microbiome (26, 27). The results indicated that only less than 1% of the species on microplastics were derived from the surrounding water. Another reason may be that this study was on the basis of soil microcosm incubation; therefore, the bacteria on microplastics had relatively regular origins, while the previous studies were both field-collecting studies. The uncertainty of the microplastic transfer may lead to minimal contribution.

10.1128/msystems.00352-22.9 FIG S5 Source and assembly of the plastisphere community. (A) Contribution of bulk soil species to the plastisphere species in BS treatments based on SourceTracker method. (B) Contribution of bulk soil species to the plastisphere species in YS treatments based on SourceTracker method. (C) Contribution of plankton bacteria to the plastisphere communities in aquatic environments. (D and E) Normalized stochasticity ratios of PE and PLA plastisphere in BS (D) and YS (E) treatments based on Bray-Curtis and Jaccard distances. Download FIG S5, PDF file, 0.3 MB. Copyright © 2022 Sun et al. 2022 Sun et al. https://creativecommons.org/licenses/by/4.0/ This content is distributed under the terms of the Creative Commons Attribution 4.0 International license.

**FIG 5:**
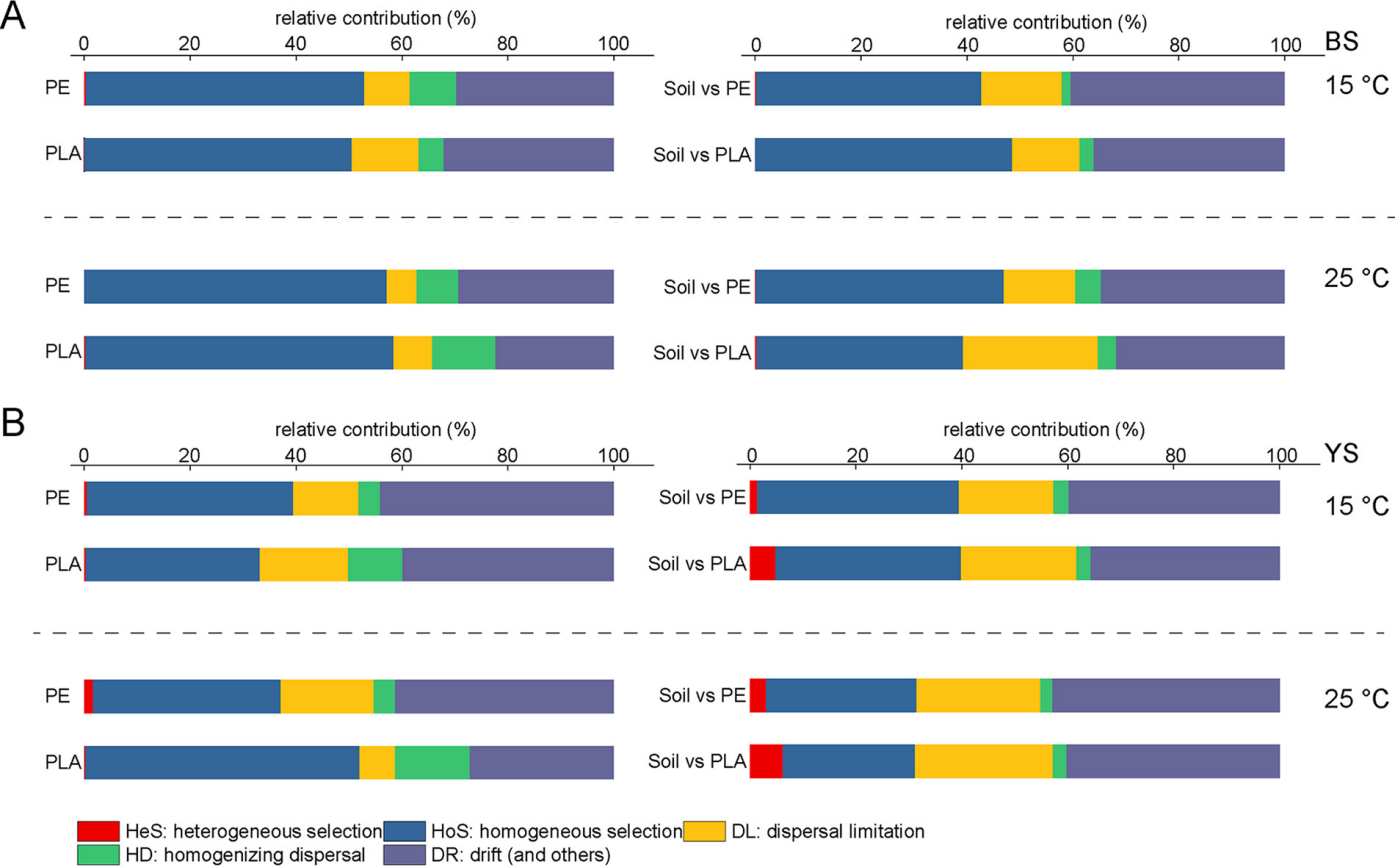
Assembly process within plastisphere and between plastisphere and bulk soil communities based on inference of community assembly mechanisms by phylogenetic bin (iCAMP). (A) Relative contribution of different processes for microbial assembly in BS treatments. (B) Relative contribution of different processes for microbial assembly in YS treatments.

The normalized stochastic ratios (NSTs) indicated that stochastic processes dominantly contributed to the assembly of plastisphere communities in soil, and higher temperature decreased their importance in structuring the plastisphere communities, especially for the PLA plastisphere (Fig. S5). For instance, in BS soil, the NST values based on Bray-Curtis and Jaccard metrics for PLA plastisphere at 15°C were 94.2% and 95.8%, respectively, which were significantly higher than those at 25°C (85.9% and 83.9%, respectively). The assembly processes were further partitioned into heterogeneous selection (HeS), homogeneous selection (HoS), dispersal limitation (DL), homogenizing dispersal (HD), and drift via the infer community assembly mechanisms by phylogenetic bin-based null model analysis (iCAMP) method (Fig. 5). In general, HoS and drift were the dominant processes in shaping plastisphere communities, ranging from 32.8 to 58.2% and 22.4 to 44.2%, respectively. Warming increased the contribution of HoS and HD and decreased the importance of drift to the assembly of plastisphere communities. Previous studies also reported that warming may increase the deterministic processes in microbial communities. Ning et al. have increased the soil temperature by experimental warming (approximately 3°C) in their grassland experimental sites, and they observed that the warming gradually enhanced HoS and decreased drift in bacterial community assembly (44). Compared to the PE plastisphere, the contribution of homogenizing processes (including HoS and HD) was higher in the PLA plastisphere. The potential reason might be that the degradation of PLA induced the importance of HoS and HD. This may be also possibly attributed to the fact that the bacteria on microplastics would gradually diffuse due to the continuous degradation and fragmentation of PLA microplastics. We also analyzed the ecological processes between the bulk soil communities and plastisphere. The contribution of dispersal limitation and heterogeneous selection was relatively high, potentially inducing the differences between soil and plastisphere communities.

As the studies’ awareness of the potential ecological risk of microplastics gradually increases, disentangling the assembly mechanisms of plastisphere communities is getting more attention. Sun et al. (27) studied the community assembly in plastisphere collected from urban rivers and one estuary, respectively, and reported that the stochastic processes critically shaped the communities on microplastics in aquatic environments (27). Through an *in situ* incubation experiment in natural coastal water, Zhang et al. found that the plastisphere microbial trajectory was mainly governed by the stochastic process (28). Similarly, studies about plastisphere in soil also demonstrated the dominant roles of stochastic processes in shaping the plastisphere microbiota. For instance, Ju et al. have found a higher proportion of stochastic processes than deterministic processes for plastisphere (45). In the current study, the NST values indicated that the stochastic processes dominantly contributed to the plastisphere communities. These limited studies may suggest the importance of ecological stochasticity for assembling the plastisphere communities. The potential reason may be attributed to these two studies being microcosm incubation experiments with narrow scales, which reduced the impact of selection and increased the role of stochastic processes (46, 47). Additionally, only considering the assembly processes within plastisphere may not be enough, as the microbes on microplastics should be originated from the surrounding environments and then form biofilms. The dispersal between plastisphere and surrounding microorganisms would also generally happen. These processes may explain how the diversity in the plastisphere is generated and maintained. The remarkable contribution of dispersal limitation and heterogeneous selection in this study suggested the isolation between the plastisphere and the bulk soil communities. What we should notice is that we only explored the processes at the end of a short incubation. The assembly processes at the initial incubation or after long-term exposure should be largely different. Further studies using field-collected or *in situ*-incubated microplastics with different scales and time intervals will be needed for a better understanding of the underlying assembly mechanisms.

In conclusion, this study advances the field by demonstrating that the plastisphere and the bulk soil communities had distinct bacterial community compositions, which were closely associated with the recruitment of special species by PE and PLA microplastics. The findings further suggested that the microbiome assembly in the plastisphere is predominantly shaped by temperature rather than by polymer type. Higher temperature may increase the divergence between the plastisphere and the soil bacterial communities. The PE plastisphere showed higher levels of microbial network complexity and stability than those in the PLA plastisphere, as the degradation products of PLA may have greater selection pressure on microbial recruitment. The assembly mechanisms further revealed the dominant roles of homogeneous selection and drift in shaping the plastisphere communities. Future studies for understanding how microbiomes transcend the ecosystem boundaries between microplastics and bulk soils will be needed.

## MATERIALS AND METHODS

### Microplastics and soils.

Biodegradable PLA and conventional PE film were purchased from the local agricultural market. The thicknesses of these two films were 15 and 10 μm, respectively. The films were cut into 5-mm by 5-mm square fragments by using a paper cutter and immersed in hexane and methanol for 7 days to discard the sorbed chemicals. The fragments were then dried in the fume hood and stored at 4°C before use.

Two unreclaimed soils, a blank clay soil (BS) from Jilin, China (43°17′N, 124°35′E) and a yellow loam sand soil (YS) from Ningxia, China (38°47′N, 106°27′E), were used in this study. We sampled three independent field replications (0 to 20 cm) for each soil type and mixed them thoroughly. The soil was air-dried at room temperature and sieved through a 2-mm mesh. The main properties of the soils can be found in Table S1 in the supplemental material.

10.1128/msystems.00352-22.1 TABLE S1 Soil physical and chemical properties. Download Table S1, DOCX file, 0.01 MB. Copyright © 2022 Sun et al. 2022 Sun et al. https://creativecommons.org/licenses/by/4.0/ This content is distributed under the terms of the Creative Commons Attribution 4.0 International license.

### Experimental setup.

The PE and PLA microfragments were sterilized in a UV Clean Bench for 30 min to minimize the potential microbial contamination. The incubation microcosms were prepared using 1-L sterilized glass jars containing approximately 400 g of soil and 2 g of microplastics. Each treatment was performed in six replicates. The concentration of microplastics (0.5% [wt/wt]) can be considered environmentally relevant for soils under great human activities following previous studies (48, 49). The soil moisture was adjusted every other day to maintain it at approximately 20% (wt/wt) during the incubation. The jars were covered with breathable films and incubated at 15 and 25°C, respectively, in the dark for 60 days. At the end of the incubation experiment, the microplastics were picked up with a sterile tweezer, their surfaces were cleaned, and they were used for DNA extraction. The bulk soil was also sampled for DNA extraction. A total of 96 soil and microplastic samples were stored at −80°C for further analysis.

### Total DNA extraction and 16S rRNA gene sequencing.

The soil DNA was extracted from approximately 500 mg of soil using the Mo Bio PowerSoil DNA isolation kit (Qiagen, Shanghai, China), and the plastisphere DNA was extracted from approximately 40 pieces of microfragments following the instructions of Mo Bio PowerWater DNA isolation kit (Qiagen, Shanghai, China). After a quality check, the DNA was used to amplify the V3-V4 region of the 16S rRNA gene with the primer pair 338F (ACTCCTACGGGAGGCAGCA) and 806R (GGACTACHVGGGTWTCTAAT). The details of PCR were following our previous studies (24, 27). After further purification, the PCR productions were sequenced in a 2 × 300-bp paired-end format using the Illumina MiSeq platform at Majorbio BioPharm Technology Co. Ltd. (Shanghai, China).

### Bioinformation analysis.

The raw 16S rRNA gene sequences were processed using the standard QIIME2 (Quantitative Insights Into Microbial Ecology, version 2020.2) pipeline (50). The reads were trimmed according to the Q30 minimum value, denoised, and clustered into amplicon sequence variants (ASVs) using the DADA2 plugin (51). The ASVs were rarefied at the same total number of reads (26,624) to eliminate the influence of differences in sequencing depth. A total of 3,852,567 high-quality sequences were obtained after quality control and were grouped into 31,337 ASVs. The taxonomy of each representative ASV was assigned according to the silva reference database (version 138) (52).

### Statistical analysis.

The alpha diversity, including Chao1 richness, Shannon diversity, Pielou’s evenness, and Faith’s phylogenetic diversity, was estimated using QIIME2. Kruskal-Wallis rank-sum tests were used to evaluate differences between different samples, and a *P* value of <0.05 was regarded as statistically significant. Principal-coordinates analysis (PCoA) based on Bray-Curtis and Jaccard dissimilarities among samples was used to visualize the beta diversity of our samples, and permutational multivariate analysis of variance (PERMANOVA) was used to test the significance by using the vegan package (53). The contributions of turnover and nestedness to the variations in bacterial communities between soil and plastisphere were calculated by using the betapart package (54). To identify the ASVs responsible for the plastisphere, the indicator species analysis was carried out with the indicspecies package (55).

To further illustrate the difference among plastisphere communities, the rRNA gene operon (*rrn*) copy number at the community level was calculated to estimate whether the community is more copiotrophic or more oligotrophic (39). To explore differences in the co-occurrence interactions among the microorganisms in plastisphere, the networks were constructed on the basis of Pearson correlations of log-transformed ASV abundances, and the correlation cutoff thresholds were determined on the basis of random matrix theory (56). The network characterizations, including network size, connectivity, cohesion, robustness, and vulnerability, were further calculated following the method in a previous study (29). To assess the origin of plastisphere bacteria from soil, the Bayesian SourceTracker method was used (57). The soil bacterial communities were set as sources, and the plastisphere communities were set as sinks. The normalized stochasticity ratio (NST) was applied to evaluate the microbial community assembly to be more deterministic (NST < 50%) or more stochastic (NST > 50%) based on Bray-Curtis (NST_bray_) and Jaccard (NST_jaccard_) dissimilarity (44). To further partition the contribution of ecological processes to the plastisphere assembly, a modified framework to quantitatively infer community assembly mechanisms by phylogenetic bin-based null model analysis (iCAMP) was carried out (58). The relative importance of deterministic (homogeneous and heterogeneous selection) and stochastic (homogenizing dispersal, dispersal limitation, and drift) processes was calculated. This method can provide an improved performance with higher precision and specificity than previous approaches, e.g., entire-community null model analysis. This analysis was performed by using the iCAMP package.

### Data availability.

The raw sequence data were deposited in Genome Sequence Archive (GSA; https://ngdc.cncb.ac.cn/search/?dbId=gsa&q=CRA006559) under accession number PRJCA007185. The data sets during and/or analyzed during the current study are available from the corresponding author upon reasonable request.
