# Supplementary material for: Deciphering the Mechanisms Shaping the Plastisphere Microbiota in Soil
Source: mSystems. 2022 Jul 26;7(4):e00352-22. doi: 10.1128/msystems.00352-22 (PMC9426546; doi:10.1128/msystems.00352-22)
Supplement: TABLE S2 [file msystems.00352-22-s0002.docx]

Table S2. Effects of niche, temperature, and polymer type on bacterial community structure in different soil incubations based on PERMANOVA.

|  | Niche | |  | Temperature | |  | Polymer | |  | |  |
| --- | --- | --- | --- | --- | --- | --- | --- | --- | --- | --- | --- |
| Bray | *R*^2^ (%) | Pr (> *F*) |  | *R*^2^ (%) | Pr (> *F*) |  | *R*^2^ (%) | Pr (> *F*) |  | | Variation (%) |
| BS  YS | 17.0  13.7 | 0.001  0.001 |  | 12.5  17.6 | 0.001  0.001 |  | 2.5  3.2 | 0.207  0.093 |  | 42.7  46.9 | |
| Jaccard |  |  |  |  |  |  |  |  |  | |  |
| BS  YS | 12.5  9.9 | 0.001  0.001 |  | 9.4  12.1 | 0.001  0.001 |  | 2.4  2.8 | 0.188  0.070 |  | 34.9  36.6 | |
