# Supplementary figures and images for: Deciphering the Mechanisms Shaping the Plastisphere Microbiota in Soil
Source: mSystems. 2022 Jul 26;7(4):e00352-22. doi: 10.1128/msystems.00352-22 (PMC9426546; doi:10.1128/msystems.00352-22)

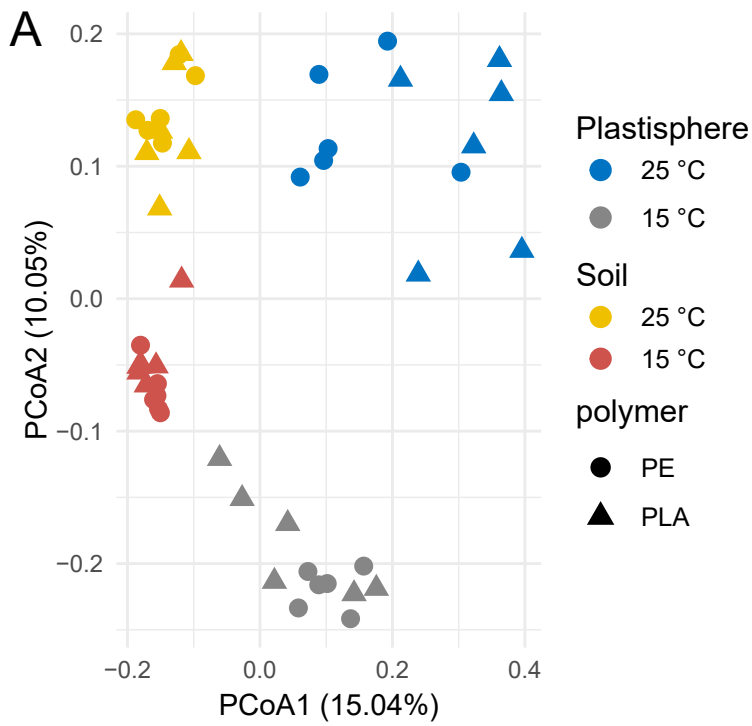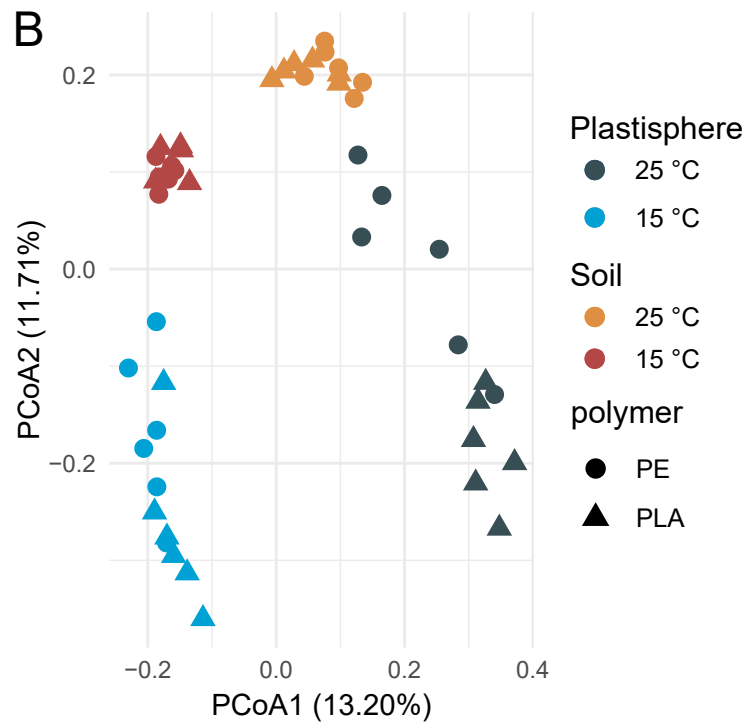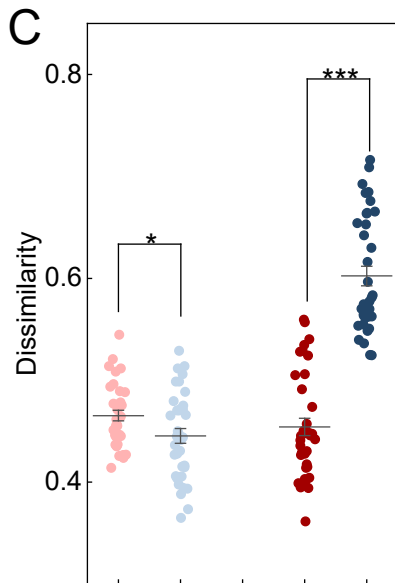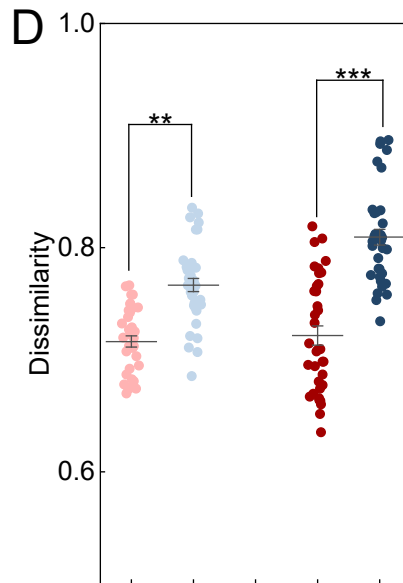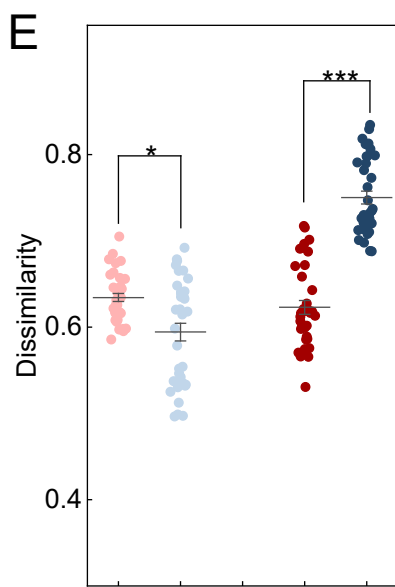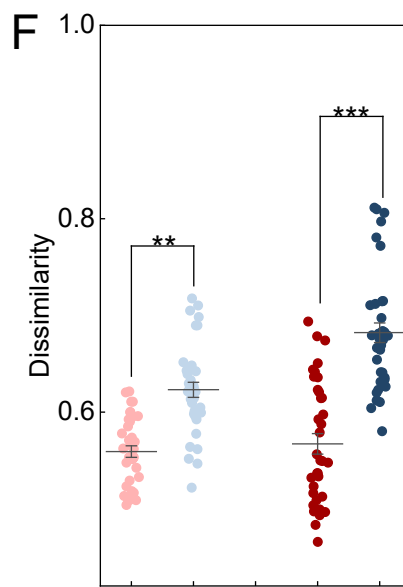

● Soil vs PE    ● Soil vs PLA    ● Soil vs PE    ● Soil vs PLA    - p > 0.05    \*\* p < 0.01  
 \* p < 0.05    \*\*\* p < 0.001

Supplement: FIG S1 [file msystems.00352-22-s0005.pdf]

A

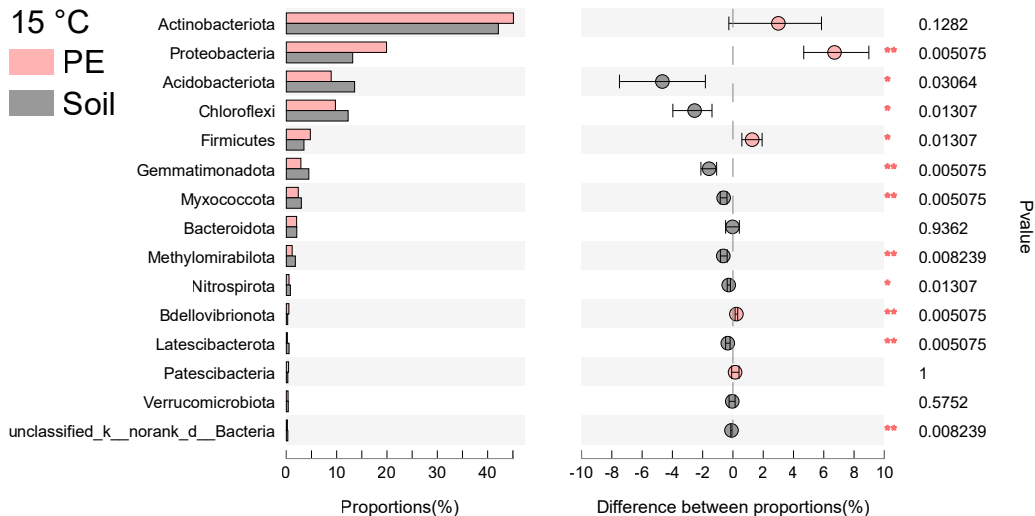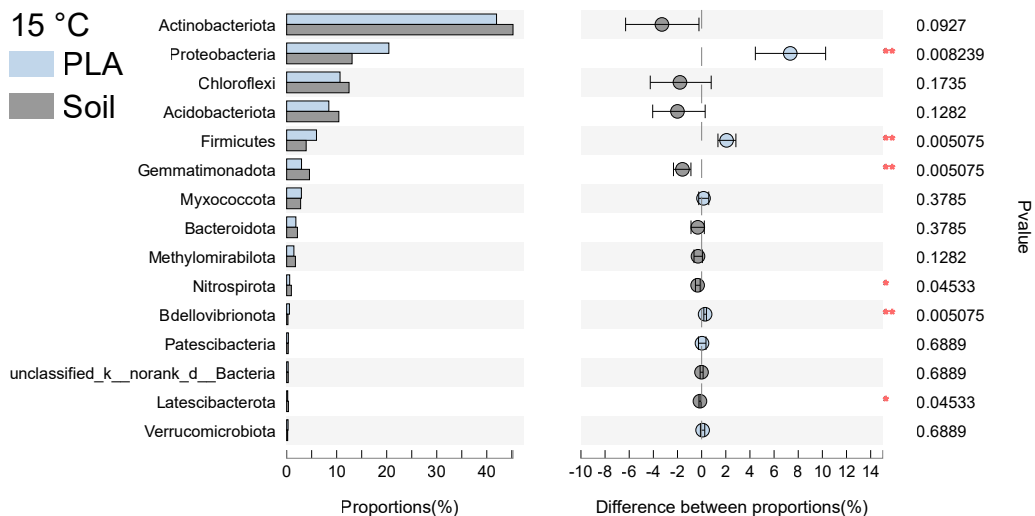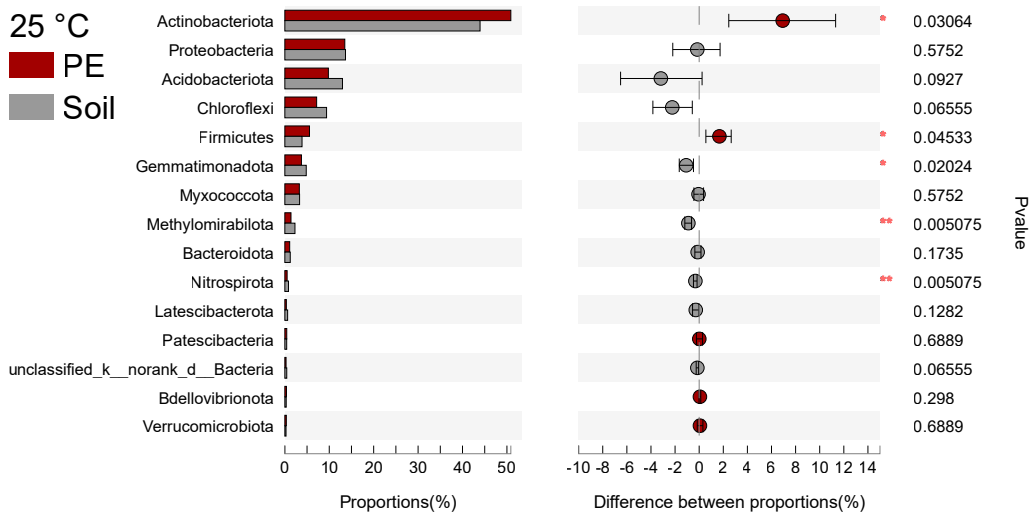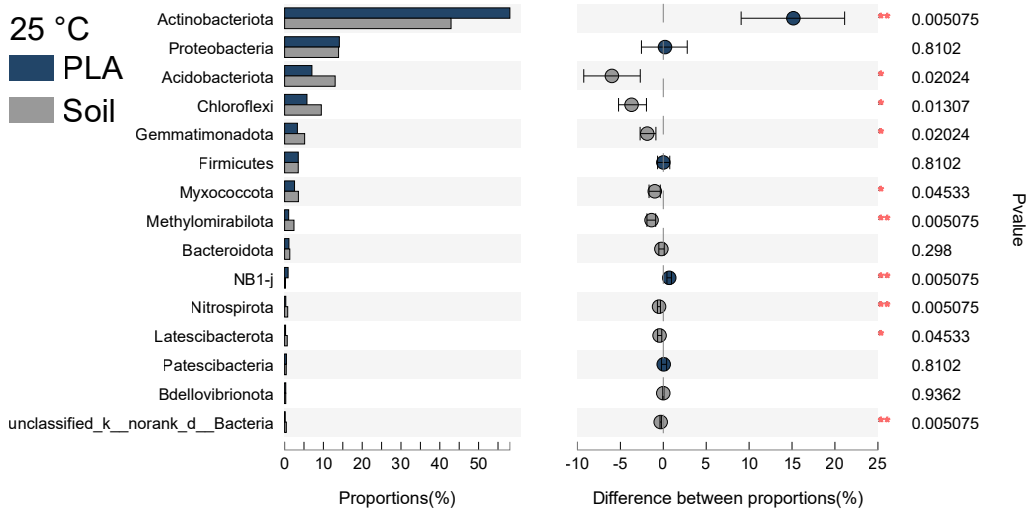

B

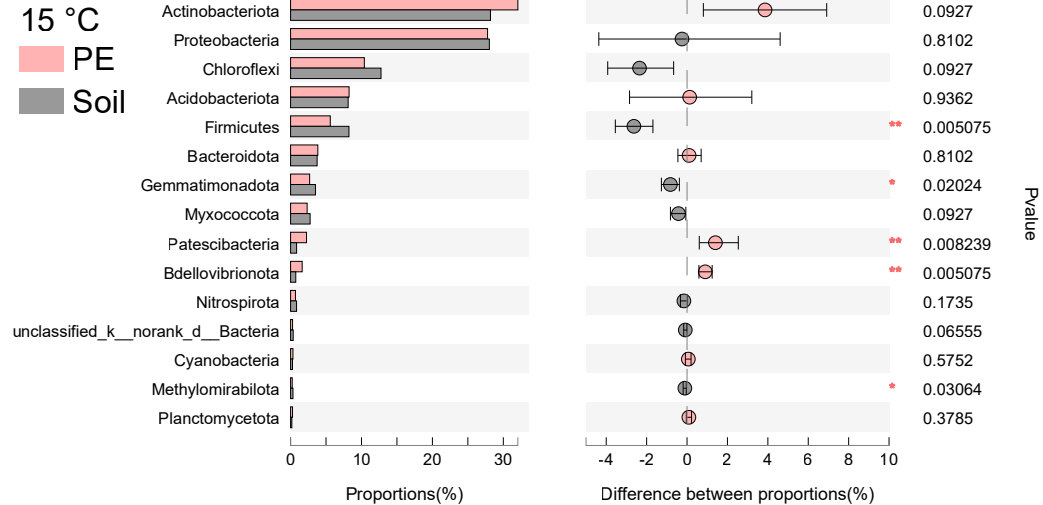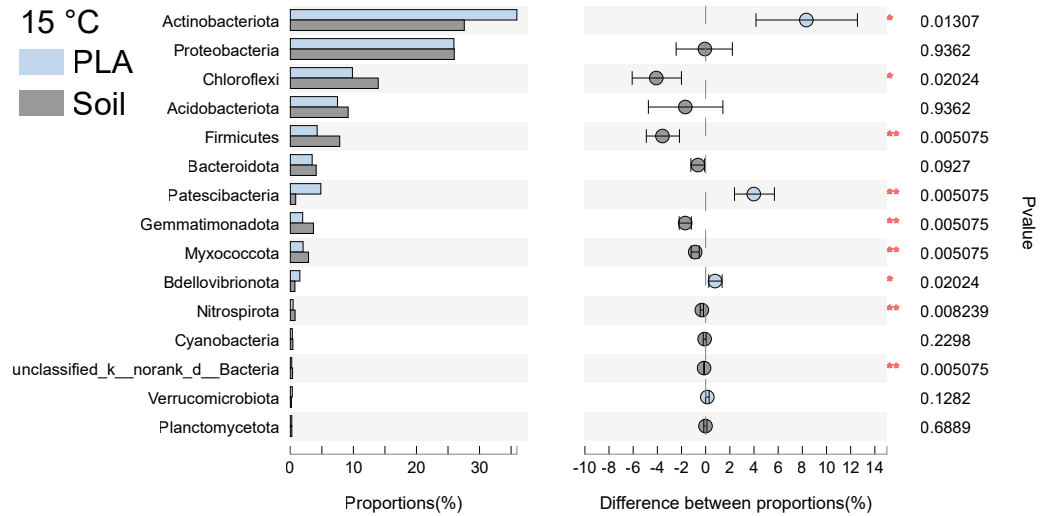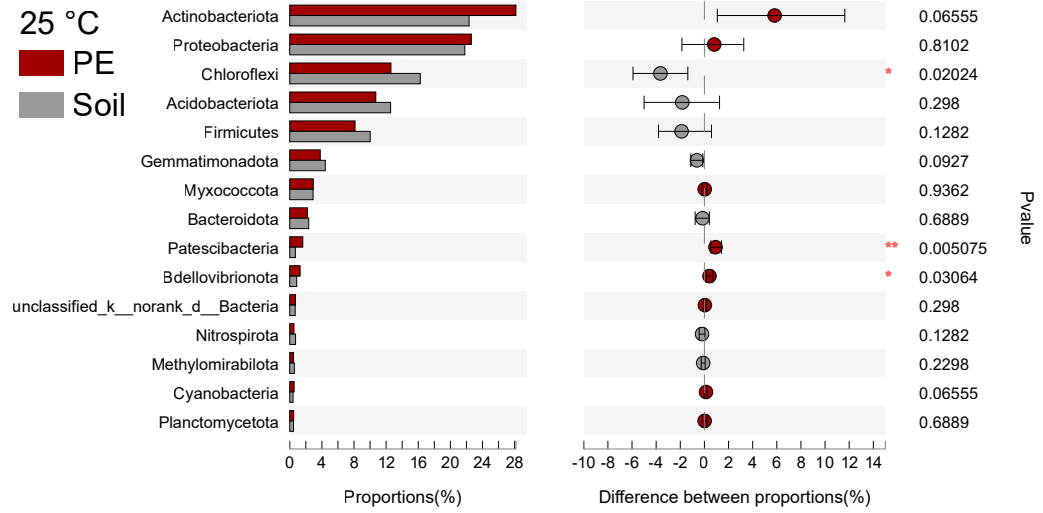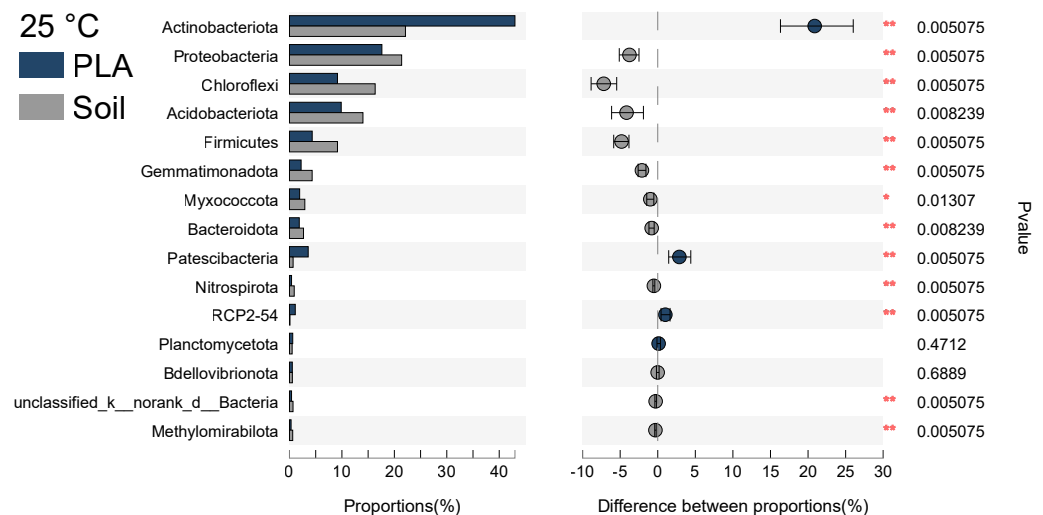

Supplement: FIG S2 [file msystems.00352-22-s0006.pdf]

A

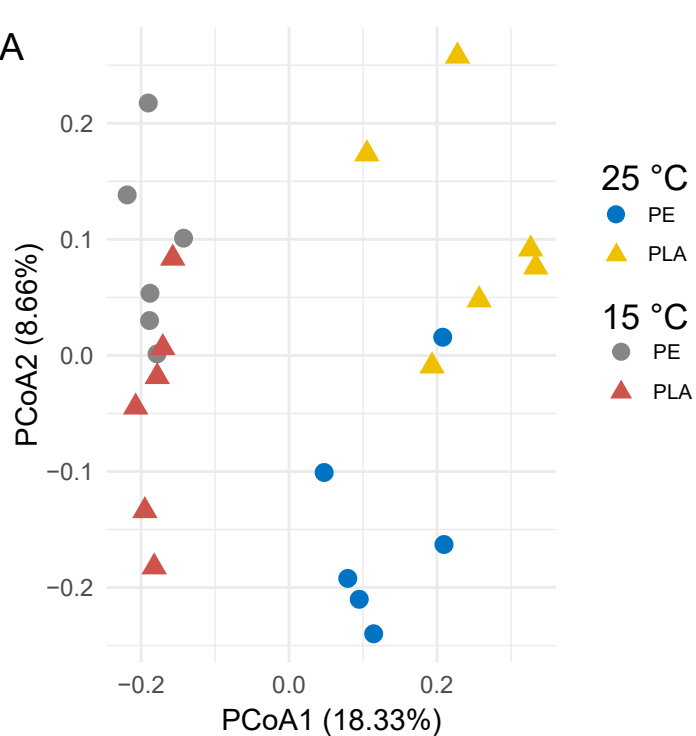

B

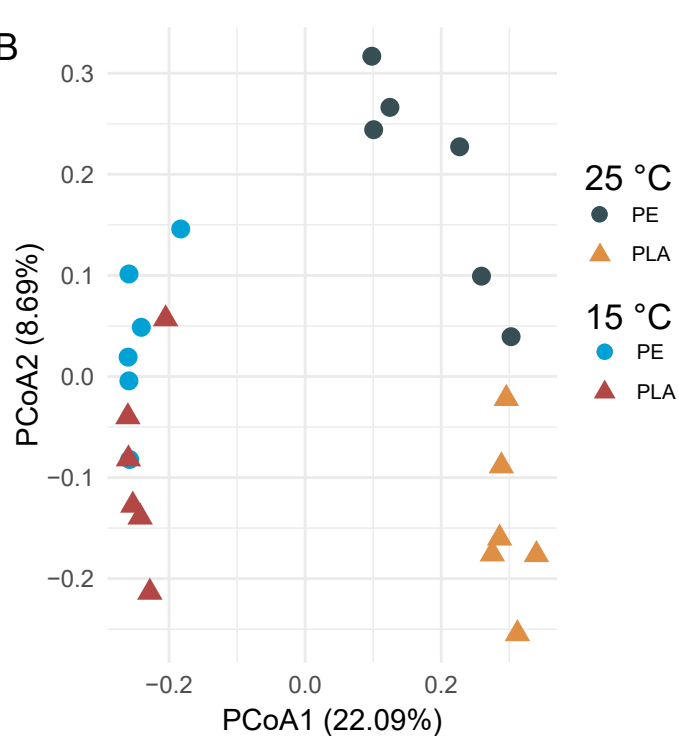

C

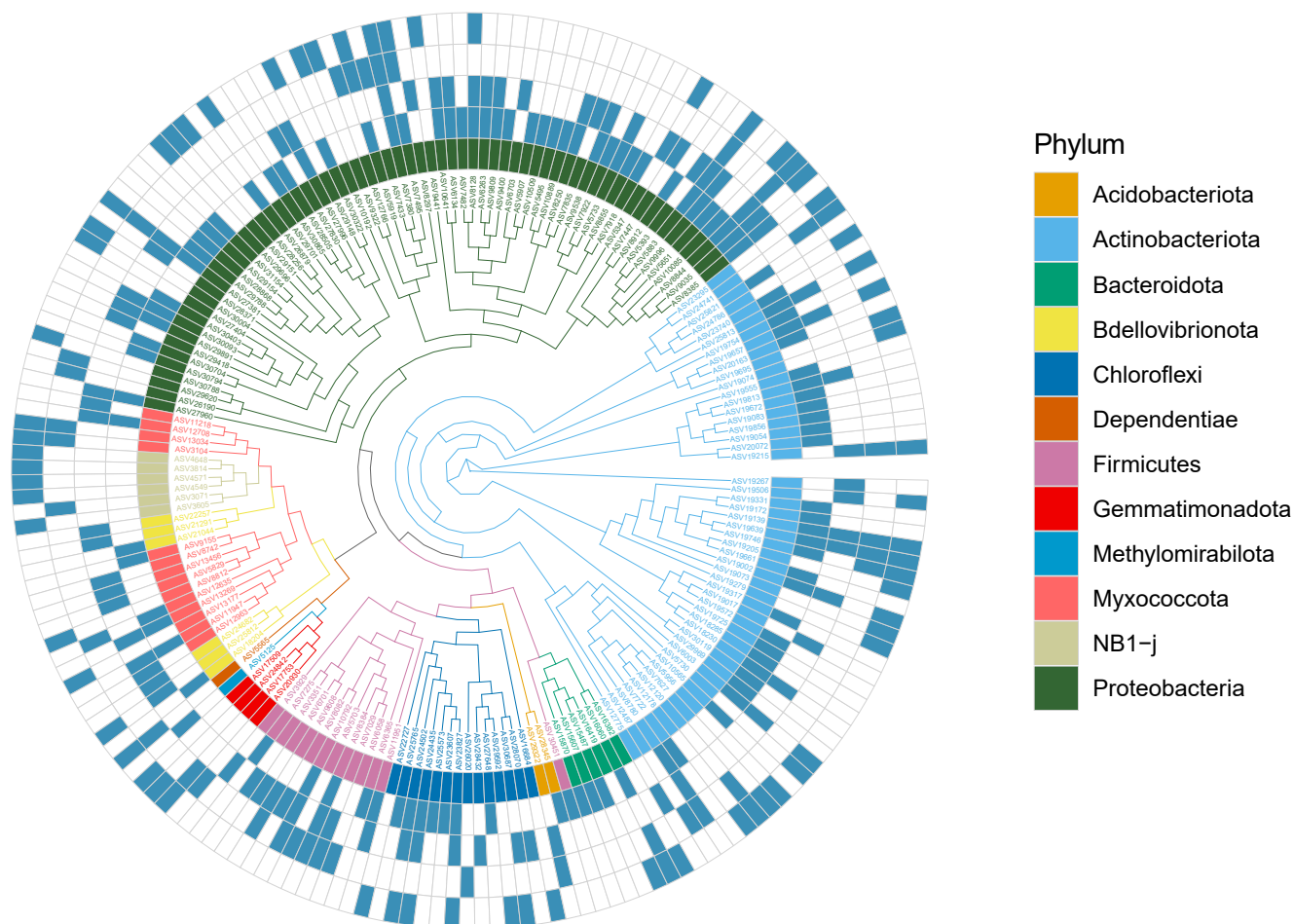

D

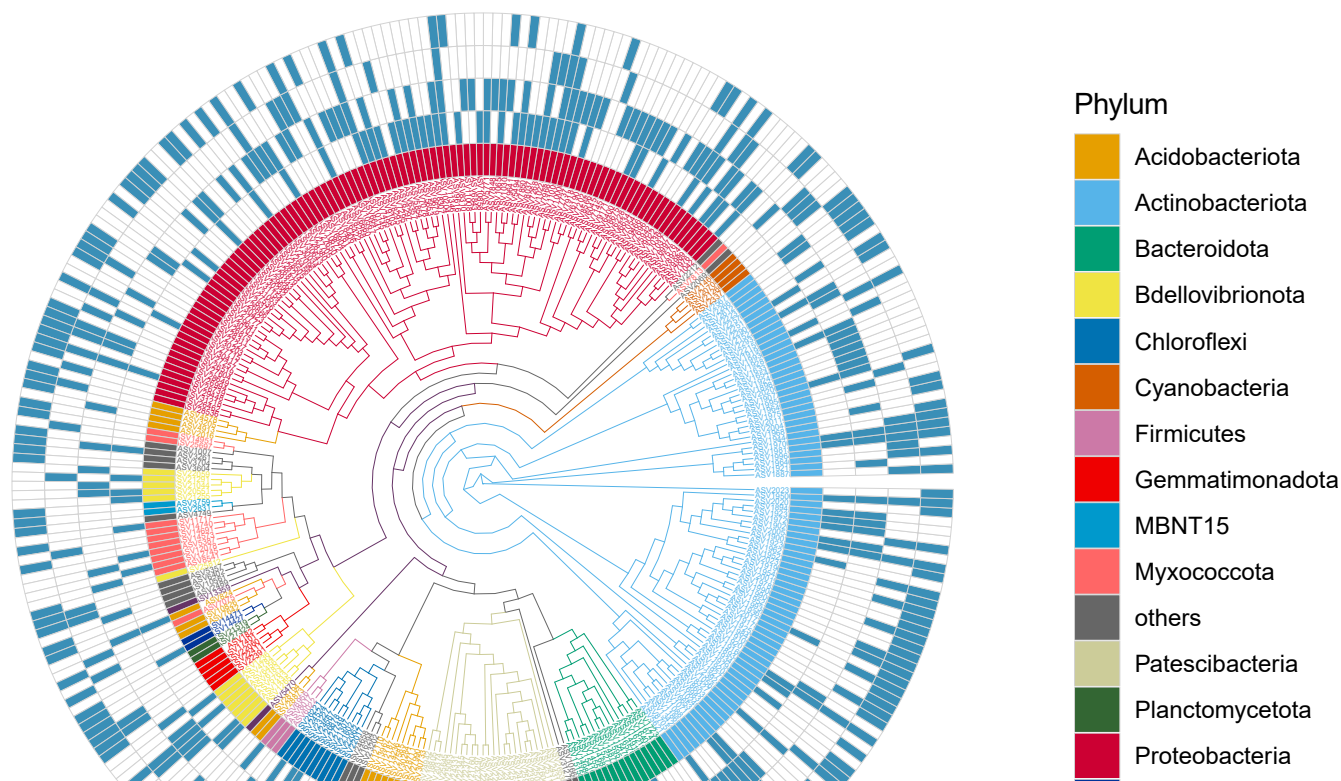

Supplement: FIG S3 [file msystems.00352-22-s0007.pdf]

**A**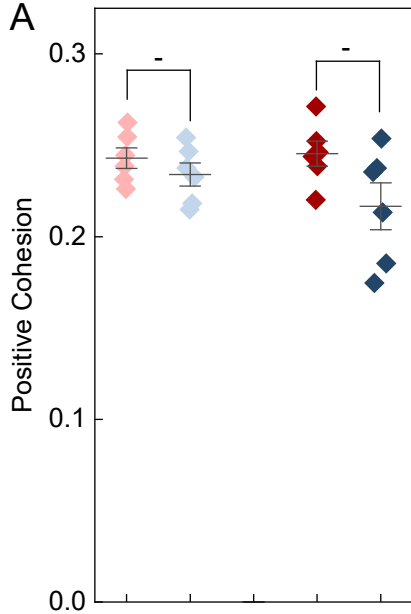**C**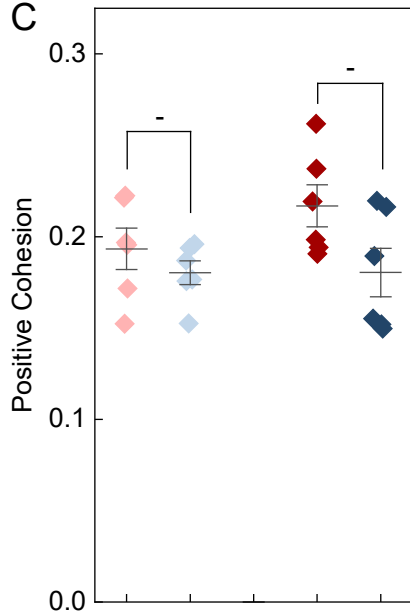**B**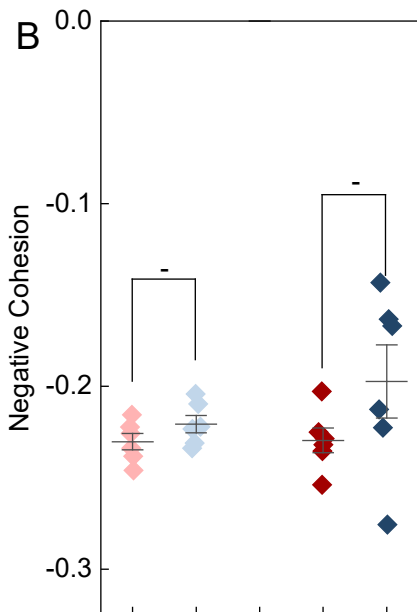**D**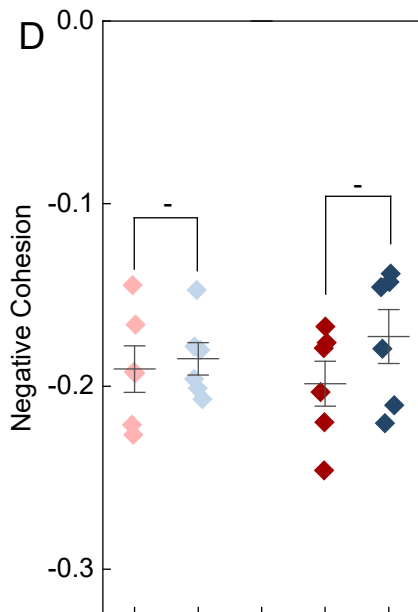

Supplement: FIG S4 [file msystems.00352-22-s0008.pdf]

**A**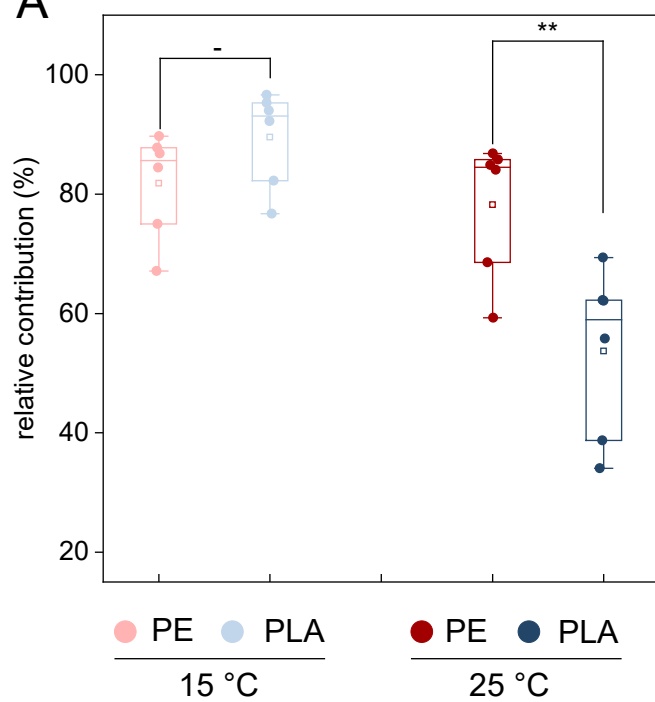**B**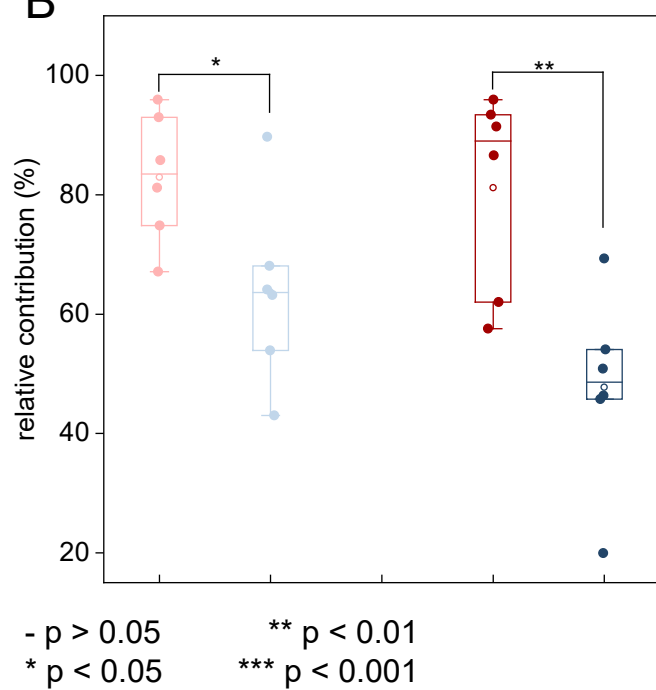**C**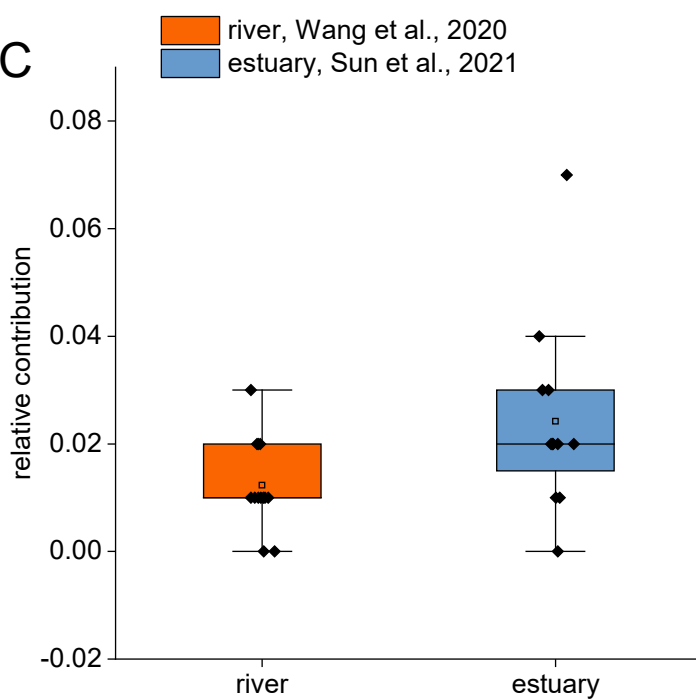**D**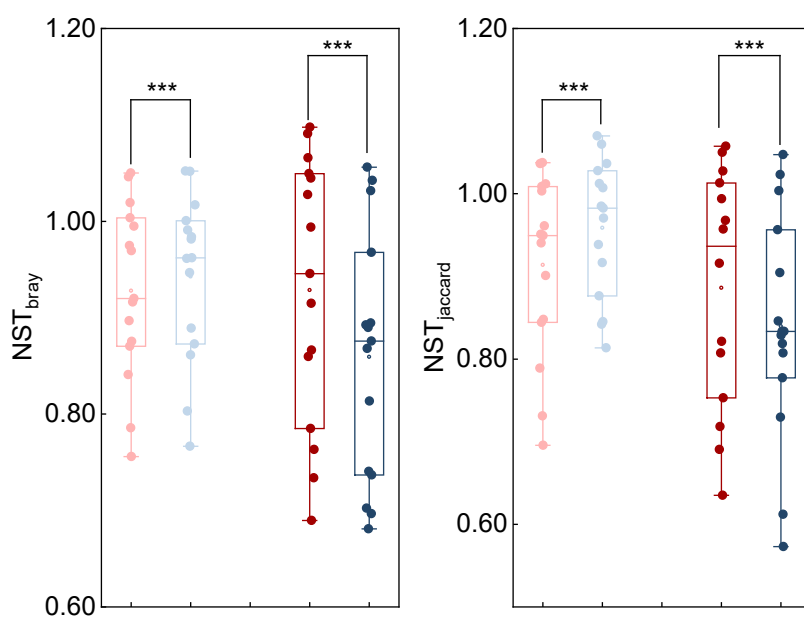**E**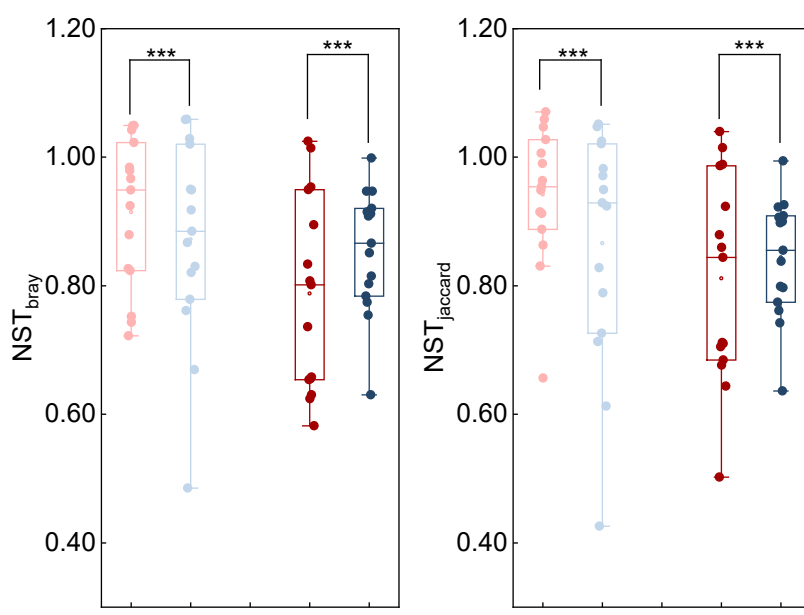

PE PLA PE PLA

15 °C 25 °C

- p > 0.05  
\* p < 0.05  
\*\* p < 0.01  
\*\*\* p < 0.001

Supplement: FIG S5 [file msystems.00352-22-s0009.pdf]
