# Supplementary material for: Deciphering the Mechanisms Shaping the Plastisphere Microbiota in Soil
Source: mSystems. 2022 Jul 26;7(4):e00352-22. doi: 10.1128/msystems.00352-22 (PMC9426546; doi:10.1128/msystems.00352-22)
Supplement: TABLE S3 [file msystems.00352-22-s0003.docx]

Table S3. Effects of temperature, and polymer type on plastisphere community structure in different soil incubations based on PERMANOVA.

|  | Temperature | | Polymer | |  |
| --- | --- | --- | --- | --- | --- |
| Bray | *R*^2^ (%) | Pr (> *F*) | *R*^2^ (%) | Pr (> *F*) | Variation (%) |
| BS  YS | 22.4  30.5 | 0.001  0.001 | 6.1  8.4 | 0.104  0.051 | 35.7  44.3 |
| Jaccard |  |  |  |  |  |
| BS  YS | 16.9  21.2 | 0.001  0.001 | 5.9  7.2 | 0.067  0.052 | 29.3  33.9 |
