# Supplementary material for: Deciphering the Mechanisms Shaping the Plastisphere Microbiota in Soil
Source: mSystems. 2022 Jul 26;7(4):e00352-22. doi: 10.1128/msystems.00352-22 (PMC9426546; doi:10.1128/msystems.00352-22)
Supplement: TABLE S4 [file msystems.00352-22-s0004.docx]

Table S4. The topological properties of plastisphere networks in BS and YS treatments.

| BS | 15 °C | | 25 °C | |
| --- | --- | --- | --- | --- |
|  | PE | PLA | PE | PLA |
| Total nodes  Total links  Negative links (%)  R square of power-law  Average degree (avgK)  Average clustering coefficient (avgCC)  Average path distance (GD)  Geodesic efficiency (E)  Harmonic geodesic distance (HD)  Centralization of degree (CD)  Centralization of betweenness (CB)  Centralization of stress centrality (CS)  Centralization of eigenvector centrality (CE)  Transitivity (Trans)  Connectedness (Con)  Modularity | 354  595  40.6  0.817  3.362  0.338  9.501  0.149  6.718  0.03  0.141  1.49  0.342  0.439  0.604  0.849 | 334  495  52.1  0.838  2.964  0.286  7.829  0.183  5.463  0.024  0.096  0.507  0.339  0.401  0.341  0.851 | 284  526  32.9  0.735  3.704  0.323  6.722  0.205  4.867  0.033  0.084  0.728  0.309  0.416  0.486  0.768 | 180  363  20.6  0.789  4.033  0.384  6.1  0.229  4.36  0.056  0.12  0.939  0.303  0.458  0.6  0.743 |
| YS | 15 °C | | 25 °C | |
|  | PE | PLA | PE | PLA |
| Total nodes  Total links  Negative links (%)  R square of power-law  Average degree (avgK)  Average clustering coefficient (avgCC)  Average path distance (GD)  Geodesic efficiency (E)  Harmonic geodesic distance (HD)  Centralization of degree (CD)  Centralization of betweenness (CB)  Centralization of stress centrality (CS)  Centralization of eigenvector centrality (CE)  Transitivity (Trans)  Connectedness (Con)  Modularity | 342  542  47.8  0.698  3.17  0.312  10.358  0.134  7.443  0.017  0.137  3.042  0.357  0.409  0.7  0.851 | 290  413  46.0  0.72  2.848  0.287  10.887  0.132  7.589  0.025  0.23  1.273  0.332  0.363  0.732  0.844 | 306  669  43.9  0.801  4.373  0.37  7.192  0.186  5.363  0.042  0.116  2.86  0.273  0.462  0.728  0.719 | 257  370  50.8  0.822  2.879  0.307  9.131  0.159  6.305  0.028  0.172  1.266  0.318  0.409  0.548  0.852 |
