# Supplementary material for: Deciphering the Mechanisms Shaping the Plastisphere Microbiota in Soil
Source: mSystems. 2022 Jul 26;7(4):e00352-22. doi: 10.1128/msystems.00352-22 (PMC9426546; doi:10.1128/msystems.00352-22)
Supplement: TABLE S1 [file msystems.00352-22-s0001.docx]

Table S1. Soil physical and chemical properties.

|  | BS | YS |
| --- | --- | --- |
| SOC (g/kg)  Total N (g/kg)  C/N ratio  pH  EC (uS/cm)  clay (%)  silt (%)  sand (%) | 15.94 ± 0.08  1.64 ± 0.07  9.71 ± 0.42  7.04 ± 0.01  68 ± 1.41  58.33  27.75  13.92 | 4.41 ± 0.04  0.94 ± 0.12  11.51 ± 0.95  9.01 ± 0.01  176.50 ± 9.19  6.60  15.32  78.08 |
